# Evaluation of binding and inhibition mechanism of dietary phytochemicals with sphingosine kinase 1: Towards targeted anticancer therapy

**DOI:** 10.1038/s41598-019-55199-3

**Published:** 2019-12-10

**Authors:** Preeti Gupta, Taj Mohammad, Rashmi Dahiya, Sonam Roy, Omar Mohammed Ali Noman, Mohamed F. Alajmi, Afzal Hussain, Md. Imtaiyaz Hassan

**Affiliations:** 1 Centre for Interdisciplinary Research in Basic Sciences, Jamia Millia Islamia, Jamia Nagar, New Delhi 110025 India; 2 Department of Pharmacognosy College of Pharmacy, King Saud University, Riyadh, 11451 Saudi Arabia

**Keywords:** Kinases, Medicinal chemistry

## Abstract

Sphingosine kinase 1 (SphK1) has recently gained attention as a potential drug target for its association with cancer and other inflammatory diseases. Here, we have investigated the binding affinity of dietary phytochemicals viz., ursolic acid, capsaicin, DL-α tocopherol acetate, quercetin, vanillin, citral, limonin and simvastatin with the SphK1. Docking studies revealed that all these compounds bind to the SphK1 with varying affinities. Fluorescence binding and isothermal titration calorimetric measurements suggested that quercetin and capsaicin bind to SphK1 with an excellent affinity, and significantly inhibits its activity with an admirable IC_50_ values. The binding mechanism of quercetin was assessed by docking and molecular dynamics simulation studies for 100 ns in detail. We found that quercetin acts as a lipid substrate competitive inhibitor, and it interacts with important residues of active-site pocket through hydrogen bonds and other non-covalent interactions. Quercetin forms a stable complex with SphK1 without inducing any significant conformational changes in the protein structure. In conclusion, we infer that quercetin and capsaicin provide a chemical scaffold to develop potent and selective inhibitors of SphK1 after required modifications for the clinical management of cancer.

## Introduction

Sphingolipid metabolites have emerged as potent mediators of important signalling cascades responsible for regulating various cellular processes. Ceramide, sphingosine and sphingosine-1-phosphate (S1P) are the key players among sphingolipid metabolites^1,2^. Ceramide and sphingosine serve as ‘pro-apoptotic’ molecules and mediate cell cycle arrest and induce apoptosis. On the other hand, S1P functions as ‘pro-survival’ molecule and promotes cell proliferation and survival^3,4^. The balance between intracellular levels of these two interconvertible and oppositely acting sphingolipid metabolites forms a “sphingolipid-rheostat” which is critical in determining the cell fate^5^. A lean of this balance towards ceramide side makes the cell destined towards apoptotic or death pathways, whereas cell growth and survival is induced when S1P accumulates within the cell^6^. Interestingly, modulation of this rheostat so as to increase the levels of ceramide or sphingosine at the cost of S1P can be adopted as a therapeutic strategy to combat cancer^7,8^.

Sphingosine kinase (SphK) is one of the key regulators of this rheostat as it generates S1P from sphingosine thereby decreasing the intracellular levels of both sphingosine and ceramide^9^. Two major isoforms of SphK has been found, SphK1 and SphK2, each having distinct intracellular location, tissue distribution and biological functions^10^. SphK1 is normally present in cytoplasm but translocated to cell membrane when activated, whereas SphK2 is found in nucleus. Notably, the two isoforms have been demonstrated to regulate diverse cellular processes. For example, SphK1 induces cell growth and proliferation, whereas SphK2 promotes cell growth arrest and apoptosis^11^.

SphK1 is activated by several agonists, including mitogens, pro-inflammatory cytokines, and diverse growth factors, which is followed by its translocation to plasma membrane. The stimulated SphK1 produces S1P, a multifunctional lipid metabolite, from sphingosine leading to transient increase in its intracellular levels^12,13^. S1P plays key role in regulating diverse biological processes which are crucial for cancer progression and inflammation such as cell proliferation, differentiation, invasion and angiogenesis^14–16^. S1P acts both extracellularly by interacting with a family of five transmembrane G-protein-coupled receptors, termed as S1P receptor 1 to 5 (S1P1-5), and via intracellular targets such as various nuclear and cytoplasmic proteins which are involved in epigenetic regulation of specific genes, cell growth and calcium homeostasis^17,18^.

Recently, SphK1 has received a great attention due to its involvement in a number of human pathologies, including cancer, rheumatoid arthritis, pulmonary fibrosis, diabetes, asthma and neurodegenerative disorders^19,20^. Over-expression of SphK1 has been observed in malignancies of various organs such as breast, lung, uterus, colon, pancreas, ovary, kidney, as well as in leukemia^21–26^. SphK1 up-regulation has also been linked to the poor prognosis of many human cancers^24^. Furthermore, SphK1 plays important role in processes like angiogenesis, tumorigenesis and chemotherapy resistance which are crucial for metastasis and cancer progression^19^. Li *et al*.^27^ has demonstrated that the over-expression of SphK1 induces NF-κB activation and shortened the cell cycle thereby promoting the proliferation of breast epithelial cells. In another study, researchers have found a strong correlation between elevated SphK1 expression levels and short survival times in patients with gastric cancer^28^.

Owing to its remarkable role in cancer progression and metastasis, and other inflammatory diseases, SphK1 presents a novel therapeutic target to develop effective therapeutics to combat these diseases. The effects of some synthetic sphingosine analogues as SphK1 inhibitors have been investigated in animal models of human diseases^29,30^. But the synthetic therapeutics are often plagued with poor water solubility, limited bioavailability, and undesirable side effects^31–33^. Interestingly, compounds derived from natural sources impart no or minimal side effects and hence can be exploited to design highly specific and potent inhibitors against drug targets^34,35^. Additionally, the chemical structure of promising natural compound can be modified strategically so as to improve their aqueous solubility, absorption and metabolism^36^. Furthermore, the anticancer, anti-inflammatory, anti-diabetic and antioxidant effects of many plant derived compounds have been very well established that further makes their candidature strong as drug molecules^37–39^. The anti-inflammatory properties of some natural compounds in mouse model of SphK1-associated diseases have also been evaluated but the mechanism through which they exert their effects has not been assessed yet^40,41^.

In the present study, we screened a series of plant-derived natural compounds to evaluate their inhibitory potential against SphK1. The interaction of compounds showing best inhibitory potential was evaluated at atomic level using molecular dynamics (MD) approach to get an insight into the binding site of compound with the SphK1, and the amino acid residues involved in the binding process.

## Materials and Methods

### Materials

Luria broth and Luria agar were purchased from Himedia. Kanamycin and Tris were purchased MP Biomedicals, LLC (France). Ni-NTA resin was procured from Thermo Scientific (USA). BIOMOL^®^ Green reagent was bought from Enzo (New York, USA). Isopropyl β-D-1-thiogalactopyranoside (IPTG), quercetin, vanillin and citral were purchased from Sisco Research Laboratories. Ursolic acid, capsaicin, DL-α tocopherol acetate and limonin were bought from Tokyo Chemical Industry Co., Ltd. (Tokyo, Japan). All reagents used were of analytical grade.

### Expression and purification of SphK1

SphK1 was successfully expressed in BL21-Gold (DE3) competent cells and subsequently purified by Ni-NTA affinity chromatography in a single step as described^42^. Briefly, the recombinant cells having plasmid with *SphK1* gene insert were grown, and protein expression was induced by 1 mM at 37 °C for 3–4 hours. The cell pellet obtained was suspended in lysis buffer (50 mM Tris, 250 mM NaCl, 20 mM EDTA 0.1 mM PMSF and 1% of Triton 100, pH 8.0) and subjected to sonication to prepare inclusion bodies. Solubilisation of inclusion bodies was done by incubating them in buffer (50 mM Tris-HCl pH 8.0, 150 mM NaCl) containing 0.5% of N-Laurousyl sarcosine for 3–4 hours at room temperature followed by centrifugation at 10,000 rpm for 40 minutes. The supernatant obtained was loaded on Ni-NTA column for binding, followed by washing with 10 mM imidazole and elution with increasing concentrations of imidazole (20 mM to 400 mM). Purity of eluted fractions collected was assessed by SDS-PAGE. Fractions showing single band of protein were pooled and dialyzed extensively against 20 mM Tris-HCl buffer (pH 8.0) containing 100 mM NaCl. Protein concentration was determined using a molar absorption coefficient of 48275 M^−1^cm^−1^ at 280 nm on Jasco V-660 UV-visible spectrophotometer.

### Molecular docking

Molecular docking and MD simulation studies were carried out on DELL^®^ workstation with Intel^®^ Xeon^®^ CPU E5-2609 v3 @ 1.90 GHz processor with 64 GB RAM and two terabyte hard disk running on Ubuntu 18.04.2 LTS operating system. GROMACS 5.1.2 package was used to perform MD simulations. Computational tools such as PyMOL^43^, VMD (visual molecular dynamics)^44^ and QtGrace were used for visualization, evaluation and analysis of MD trajectories.

Atomics coordinates of SphK1 structure were taken from the Protein Data Bank (PDB ID: 3VZB), and the structure of quercetin was downloaded from the PubChem database and processed in MGL tools^45^. AutoDock Vina^46^ was used for docking purpose. PyMOL and Discovery Studio Visualizer^47^ were employed to visualize the structures for the analysis of bound conformation and different interactions between quercetin and SphK1.

### MD simulations

MD simulations were carried out for 100 ns on free SphK1 and SphK1-quercetin docked complex at 300 K of molecular mechanics level using GROMOS96 43a1 force-field in GROMACS 5.1.2. The structural coordinates of SphK1 were downloaded from the Protein Data Bank (PDB) with PDB ID: 3VZB and processed in SPDBV. The topology and force-field parameters for quercetin were generated from the PRODRG server and merged into the parent file of SphK1 to make complex. Both the systems were soaked in a 10 Å dimension sized cubic box for solvation in the SPC216 solvent model and were neutralized using counterions. Energy minimization was carried out using 1500 steps of steepest descent to remove bad contacts in the solvated systems. The temperature of both the systems was then raised up gradually from 0 K to 300 K during the equilibration time of 100 ps at constant volume, pressure (1 atm) and temperature (300 K) under periodic boundary conditions. The final MD run was set to 100,000 ps for both systems, and resulting trajectories were saved for further analysis using inbuilt utilities of GROMACS such as *gmx energy*, *gmx rms*, *gmx rmsf*, *gmx gyrate*, *gmx sasa* and *gmx sham*. A detailed description of MD simulations is reported elsewhere^48–52^.

We also performed the principal component (PC) and free energy landscape (FEL) analysis of SphK1 before and after quercetin binding. These methods employ the calculation and diagonalization of the covariance matrix which can be calculated as:

$$\begin{array}{ll}{C}_{ij}=\langle ({{\rm{x}}}_{i}-\langle {{\rm{x}}}_{i}\rangle )\,({{\rm{x}}}_{j}-\langle {{\rm{x}}}_{j}\rangle )\rangle  & (i,\,j=1,\,2,\,3\,\ldots ,\,3{\rm{N}})\end{array}$$

here, x_*i*_ = x_*j*_ is the coordinate of the *i*^th^/*j*^th^ atom of the system, whereas 〈−〉 represents an ensemble average. FELs were also constructed for both systems to understand the stability, folding and function behaviour of SphK1 before and after quercetin binding. The FEL can be constructed as:

$${\Delta }G(X)=-\,{K}_{B}T\,\mathrm{ln}\,P(X)$$

where *K*_B_ and *T* are the Boltzmann constant and absolute temperature, respectively. *ΔG*(*X*) is the probability distribution of the molecular system along the PCs.

### Fluorescence binding studies

Fluorescence quenching experiments were carried out on Jasco spectroflourimeter (FP-6200) using 5 mm quartz cuvette. The temperature was maintained at 25 ± 0.1 °C by external thermostated peltier device. Stock solution of ligands was prepared in DMSO and diluted to a working concentration of 50 µM in Tris buffer just prior to taking measurements. Protein solution (4 µM) was titrated with increasing concentrations of ligand and the fluorescence emission spectrum was recorded in the range of 300–400 nm by keeping the excitation wavelength constant at 280 nm. The excitation and emission slit widths were kept at 10 nm. The final spectra were obtained by subtracting with the corresponding blank. For data analysis, fluorescence intensity at λ_max_ was plotted against [ligand, μM] and then modified Stern-Volmer equation (Eq. 1) was used to derive binding parameters viz., binding constant (*K*_a_) and number of binding sites (*n*) per molecule of protein for ligand-SphK1 system.

1 $$\log ({{\rm{F}}}_{0}-{\rm{F}}/{\rm{F}})=\,\log \,{{K}}_{{\rm{a}}}+n\,\log \,[{\rm{Q}}]$$

### Isothermal titration calorimetry

Binding affinity of SphK1 to quercetin was determined by using VP-ITC microcalorimeter from MicroCal, Inc (GE, MicroCal, USA). Protein sample was prepared by extensively dialyzing it against Tris buffer (20 mM Tris-HCl, pH 8.0 and 100 mM NaCl). Stock solution of compound was diluted in the last dialyzing buffer to avoid any baseline error. Equal amount of DMSO (1.0% v/v) was added to the protein solution to prevent signal stability problems during ITC measurements. 15 µM SphK1, which was kept in the sample cell with an effective volume of 2 ml, was titrated with 750 µM of compound filled in the titration calorimetry syringe. A programmed titration was performed with a first false injection of 2 µl followed by 24 successive injections of 10 µl ligand each at 300 seconds interval. The temperature of the sample and reference cell was isothermally maintained at 25 °C, and the syringe was stirred at 307 rpm during the experiment. Heat of dilution of compound into buffer solution (no protein) was also measured and subtracted from the protein’s titration data. The binding isotherm obtained was fitted with in-built origin ‘binding site’ model to derive the thermodynamic parameters viz., stoichiometry of binding (*n*), enthalpy change (Δ*H*) and association constant (*K*_a_).

### Enzyme inhibition assay

ATPase activity of purified SphK1 was checked by malachite green-based (BIOMOL^®^ Green reagent, Enzo Life sciences) microtitre-plate assay. Fixed (2 µM) amount of protein was incubated with assay buffer (20 mM Tris-HCl, pH 8.0 and 100 mM NaCl) containing 10 mM MgCl_2_ and increasing concentrations of ATP for 30 minutes at 25 °C. The reaction was terminated by the addition of malachite green reagent followed by incubation for 20 minutes for colour development. The absorbance of each well was measured at 620 nm on a multiplate ELISA reader. Standard phosphate curve was prepared as described by manufacturer’s protocol and used to quantity the amount of free inorganic released from ATP by kinase activity.

Thereafter, ATPase inhibition assay for SphK1 was performed in the presence of compounds. Firstly, Protein (2 µM) was pre-incubated with increasing concentrations of ligand at room temperature for 60 minutes in 96-well plate. 10 mM MgCl_2_ and 150 µM freshly prepared ATP was then added to the reaction mixture and incubated for 30 minutes at 25 °C. To terminate the reaction, BIOMOL^®^ reagent was added and incubated for 20 minutes followed by absorbance measurement at 620 nm. The amount of free inorganic released was determined with the help of standard phosphate curve as described^53^. All the measurements were performed in triplicates.

## Results

### Expression and purification of recombinant SphK1

The recombinant his-tagged SphK1 was expressed in BL21 Gold (DE3) cells by IPTG induction. The protein was solubilised from inclusion bodies using N-Laurousyl sarcosine followed by its purification using Ni-NTA affinity chromatography in single step. The purity of SphK1 was evaluated by SDS-PAGE which showed a single protein band at ∼45 kDa (Fig. S1A). UV-absorption, far-UV circular dichroism and intrinsic fluorescence spectra of purified SphK1 reveal a proper folding of the recombinant protein without any aggregation (Fig. S1B–D). We analyzed the far-UV CD spectra of SphK1 to estimate the percentage of secondary structure elements in the native structure. SphK1 has 27% α-helix and 29% β-sheet which is close to the secondary structural content as reported in the crystal structure (PDB ID: 3VZB). The enzymatic activity of recombinant protein was further checked by ATPase assay suggesting the good quality of purified protein (Fig. S2). Overall, the results indicate that we have successfully expressed and purified recombinant SphK1 from *E. coli* in its biologically active native form.

### Molecular docking studies of natural compounds

We have screened a series of natural compounds including quercetin, ursolic acid, capsaicin, DL-α tocopherol acetate, citral, limonin, vanillin and simvastatin for their possible interaction with SphK1 using molecular docking approach. Molecular docking helps us to analyze the binding pattern of each compound with SphK1 that further supports in identifying the interacting residues and calculating binding affinity. Binding energy estimated from the docking results of SphK1 with various ligands is shown in Table S1. The calculated binding affinities (***ΔG***) values were estimated as −8.2 and −8.1 kcal/mol for quercetin and capsaicin, respectively with SphK1.

### Fluorescence measurements

To validate our docking results, the binding affinity of selected natural compounds with SphK1 was calculated using fluorescence measurements. A number of molecular interactions, including formation of an excited charge-transfer complex, intersystem crossing to the triplet state, molecular rearrangements, and ground-state complex formation between the fluorophore and quencher can lead fluorescence quenching^54^. SphK1 has 6 tryptophans that act as fluorophore and hence can be exploited for the ligand binding studies using fluorescence quenching approach. 4 µM of SphK1 was titrated by the successive addition of selected natural compounds from a 1.0 mM stock solution of ligands. The intrinsic fluorescence spectra were then collected in the range of 300–400 nm by keeping the excitation wavelength at 280 nm. The concentration of ligands was varied from 0 to 50 µM to obtain the saturation point.

**Figure 1:**
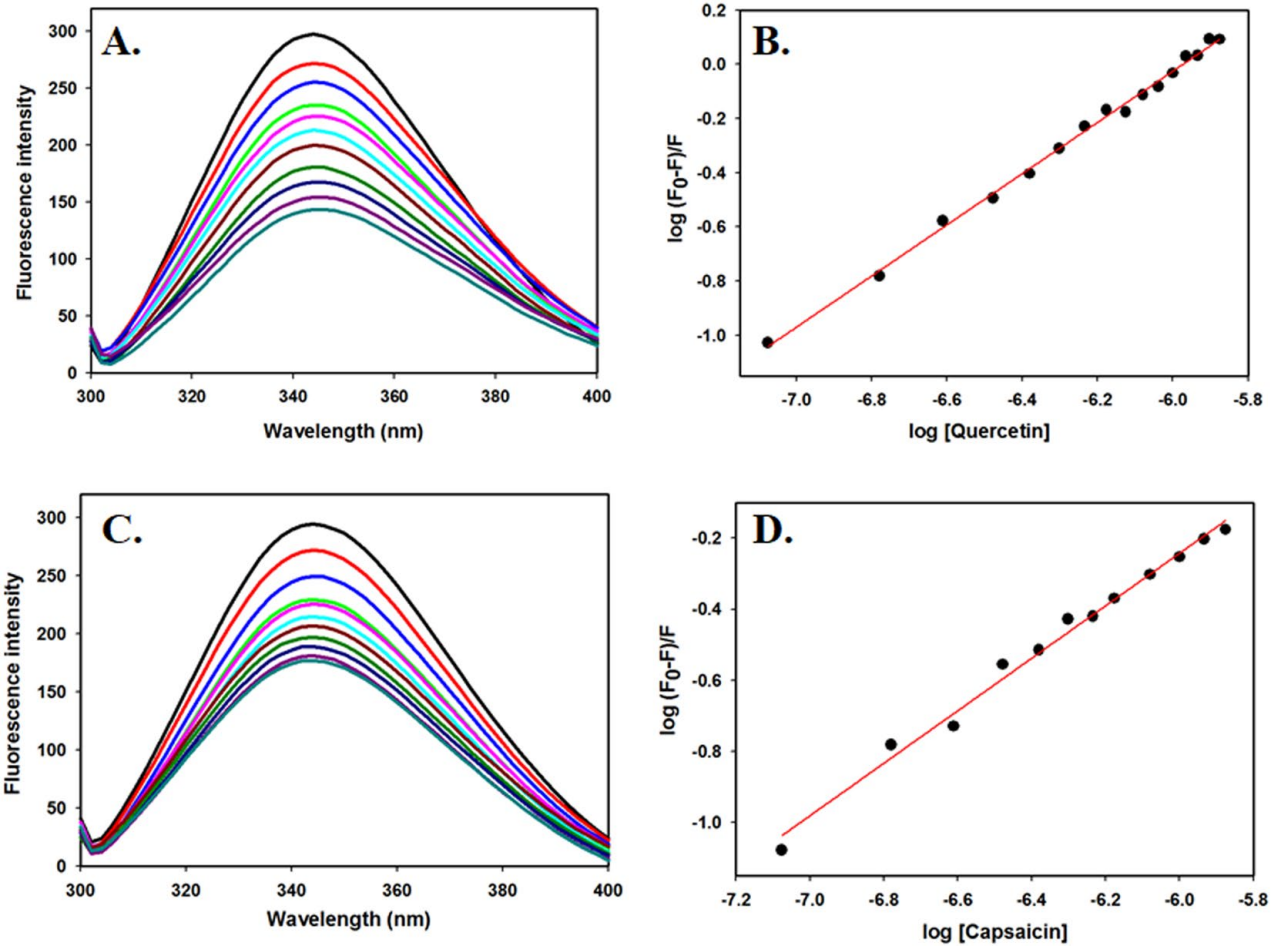
Fluorescence binding studies of SphK1 with quercetin and capsaicin. (**A**) Fluorescence spectra of SphK1 with increasing concentrations of quercetin (0–2 µM). (**B**) Modified Stern-Volmer plot (SV plot) showing fluorescence quenching of SphK1 by quercetin. (**C**) Fluorescence spectra of SphK1 with increasing concentrations of capsaicin. (**D**) Modified SV plot showing fluorescence quenching of SphK1 by capsaicin. Protein was excited at 280 nm and emission spectra were recorded in the range of 300–400 nm. The SV plot was used to calculate binding constant (*K*_a_) and number of binding sites (*n*).

Figures 1 and S3 shows the fluorescence emission spectra of SphK1 with increasing concentrations of ligands. For the compounds having good binding interaction with SphK1, a remarkable decrease in Trp fluorescence was observed with every titration. Compounds exhibiting high binding affinity with the protein saturates at lower concentration during titration whereas others with no significant quenching effects do not bind to the protein appreciably even at higher concentrations. The quenching data was analyzed using modified Stern-Volmer equation to derive binding constant (*K*_a_) and number of binding sites per SphK1 molecule (*n*). Table S1 shows the binding parameters of all studied natural compounds under investigation with SphK1. Among all the screened compounds, quercetin and capsaicin showed the best binding with SphK1 having *K*_a_ values of 4.38 × 10^5^ M^−1^ and 1.53 × 10^4^ M^−1^, respectively (Fig. 1 and Table 1). Other compounds do not show appreciable amount of fluorescence quenching and in-fact some of them induce large structural perturbations in SphK1 (Fig. S2). Hence these compounds were excluded from the studies done further to identify the potent inhibitors against SphK1.

**Table 1:** Binding and enzyme inhibition parameters of SphK1 with quercetin and capsaicin.

Compound	*Number of binding sites (*n*)	*Binding constant *K*_a_, M^−1^	Dissociation constant *K*_D_, μM	^¥^IC_50_ (µM)
Quercetin	1	4.38 × 10^5^	2.28	2.8
Capsaicin	1	1.53 × 10^4^	65.36	27.0

Values obtained from *Fluorescence and ^¥^Enzyme inhibition studies.

### ITC measurements

**Figure 2:**
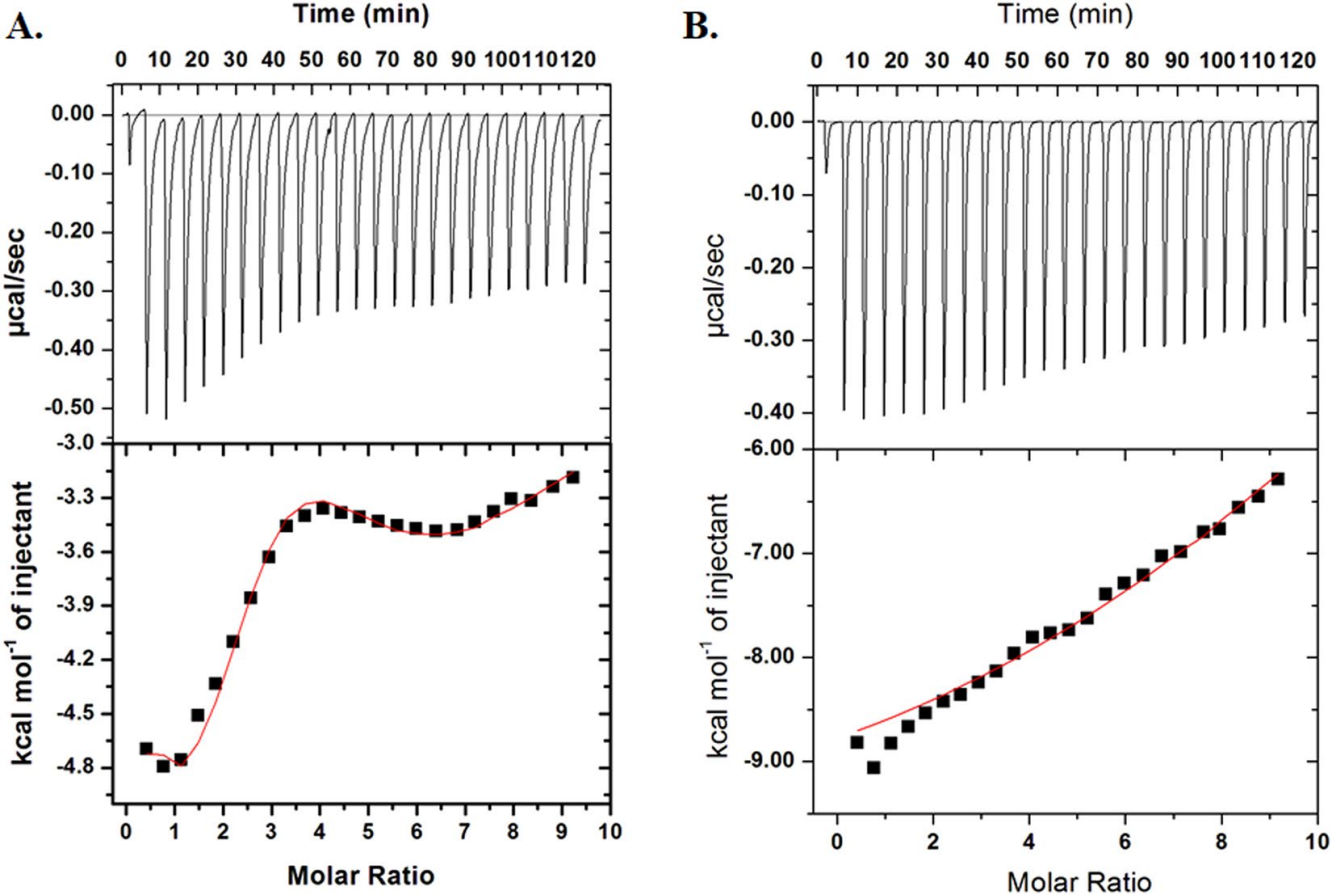
ITC measurements for the titration of SphK1 with (**A**) Quercetin and (**B**) Capsaicin. The upper panel shows the raw data points for heat produced with time with each titration of 750 µM of compound with 15 µM SphK1. The lower panel shows the binding isotherm obtained after integration of peak area and normalization to yield a plot of molar enthalpy change against each compound/SphK1 ratio. The fit curve is shown in red color line.

Fluorescence binding studies indicate that quercetin and capsaicin interact with high binding affinity to SphK1. To further validate the binding interaction, we performed ITC measurements and calculated the thermodynamic parameters viz., binding affinity, enthalpy change (*ΔH*), entropy change (*ΔS*), and stichiometry associated with SphK1-ligand binding reaction. Figure 2 shows the ITC isotherm generated from the titration of quercetin and capsaicin with SphK1 at 25 °C. The upper panel of binding isotherm with negative pulses of heat suggests that the interaction of SphK1 with quercetin and capsaicin is exothermic in nature yielding favourable values of binding enthalpy (Fig. 2 and Table 2). The lower panel shows the amount of heat released with each successive injection as a function of molar ratio of SphK1 and the compound. The blank titration measuring the heat of dilution of compound into Tris buffer was also performed. The heat associated with the addition of compound into buffer was subtracted from the heat changes by the titration of compound with protein. The corrected heat for the interaction of compound with SphK1 was plotted against the molar ratio of SphK1/Compound. The thermodynamic parameters presented in Table 2 for SphK1-ligand interaction were obtained by fitting the ITC isotherm with in-built binding model in origin software. The isotherm for SphK1-Quercetin interaction seems to be biphasic and would therefore be suggestive of the cooperative mode of interaction. The raw data was fitted to the sequential binding mode with 2, 3, 4, 5 and 6 binding sites. On comparing the chi square values for different binding modes, it was observed that the data fits best with 4 sequential binding sites having least chi square value. In contrast, SphK1-Capsaicin binding isotherm fit well with the single binding site model (Table 2). *K*_D_ values estimated for both quercetin and capsaicin lays in micromolar range that correlate well with our fluorescence binding results.

**Table 2:** Thermodynamic parameters obtained from the interaction of quercetin and capsaicin with SphK1 by ITC experiment.

Compounds	Number of binding sites, N	Association constant *K*_a_, M^−1^	Dissociation Constant *K*_D_, μM	Enthalpy Change Δ*H*, cal/mol	Δ*S*, cal/mol/deg
Quercetin	4	*K*_a1_ = 5.89 × 10^4^ ± 5.0 × 10^3^	*K*_D1_ = 16.9	Δ*H*_1_ = −5204 ± 435	Δ*S*_1_ = 4.37
		*K*_a2_ = 2.36 × 10^5^ ± 8.0 × 10^3^	*K*_D2_ = 4.2	Δ*H*_2_ = −9883 ± 696	Δ*S*_2_ = −8.57
		*K*_a3_ = 1.06 × 10^4^ ± 4.0 × 10^2^	*K*_D3_ = 94.3	Δ*H*_3_ = 3829 ± 2.18 × 10^3^	Δ*S*_3_ = 32.3
		*K*_a4_ = 7.98 × 10^3^ ± 5.9 × 10^2^	*K*_D4_ = 10.7	Δ*H*_4_ = −9289 × 10^4^ ± 4.11 × 10^3^	Δ*S*_4_ = −294
Capsaicin	1	1.02 × 10^5^ ± 6.62 × 10^4^	9.8	−1.00 × 10^4^ ± 727.6	−10.8

### Enzyme inhibition assay

**Figure 3:**
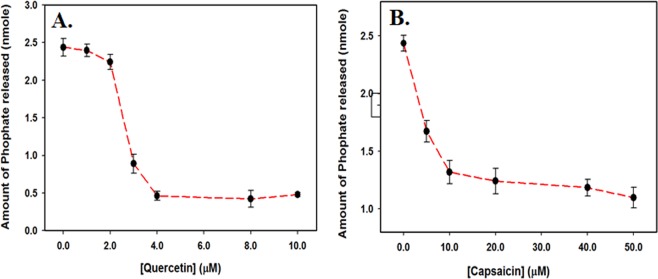
ATPase inhibition assay. ATPase inhibition as depicted by the amount of phosphate released in the presence of increasing concentrations of (**A**) Quercetin and (**B**) Capsaicin. Each measurement was performed in triplicates.

SphK1 possess ATPase activity that can be exploited to check the inhibitory potential of natural compounds. ATPase inhibition assay was performed to evaluate the efficacy of quercetin and capsaicin (the best binding compounds) in diminishing the functional activity of the enzyme. Figure 3 shows the amount of inorganic phosphate released by SphK1 in the presence of increasing concentration of quercetin and capsaicin. It is quite clear that both quercetin and capsaicin are effective in inhibiting the enzymatic activity of SphK1 at micromolar concentrations (Fig. 3B,C). Quercetin and capsaicin showed an IC_50_ value (50% of ATPase inhibition) of ~2.8 µM and ~ 27.0 µM, respectively (Table 1). Overall, our activity results suggest that quercetin and capsaicin functions as potential inhibitors of SphK1 in micromolar range.

### Molecular docking of quercetin with SphK1

**Figure 4:**
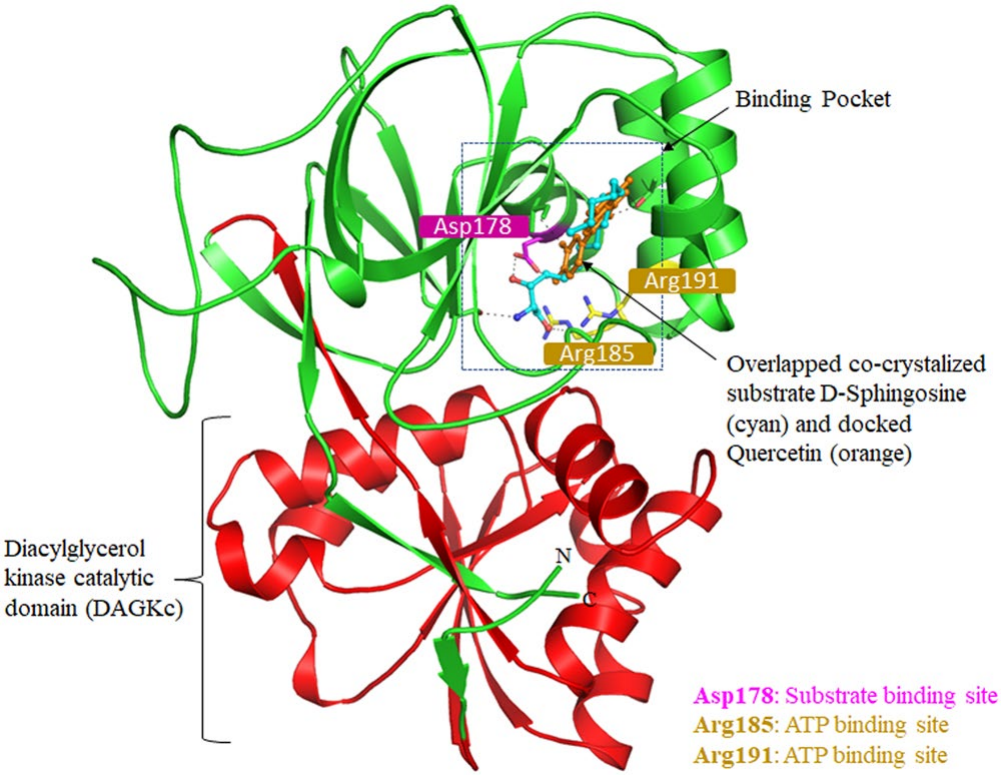
Structural organization of SphK1. Cartoon representation showing the co-crystallized ligand D-Sphingosine (cyan) and docked quercetin (orange) in the active site cavity of SphK1.

Quercetin was found to be most lethal for SphK1 activity and displayed the best binding affinity among all the natural compounds screened through a series of experiments, hence we performed docking study to get mechanistic details of SphK1-quercetin interaction at a molecular level. Molecular docking helps to predict a preferred orientation and binding prototype of a compound at the receptor’s binding pocket. In the analysis of SphK1-quercetin docked complex, we found that quercetin shows appreciable binding affinity (−8.2 kcal/mol) (Table S1), and preferentially occupy the substrate-binding pocket of SphK1. Quercetin is binding with several polar interactions and placed in the deep cavity of the SphK1 binding pocket (Fig. 4). During interaction analysis, we found that quercetin is present at the same place and mimicking the position where co-crystallized substrate D-sphingosine is present, which might presumably decreases the substrate accessibility of SphK1.

**Figure 5:**
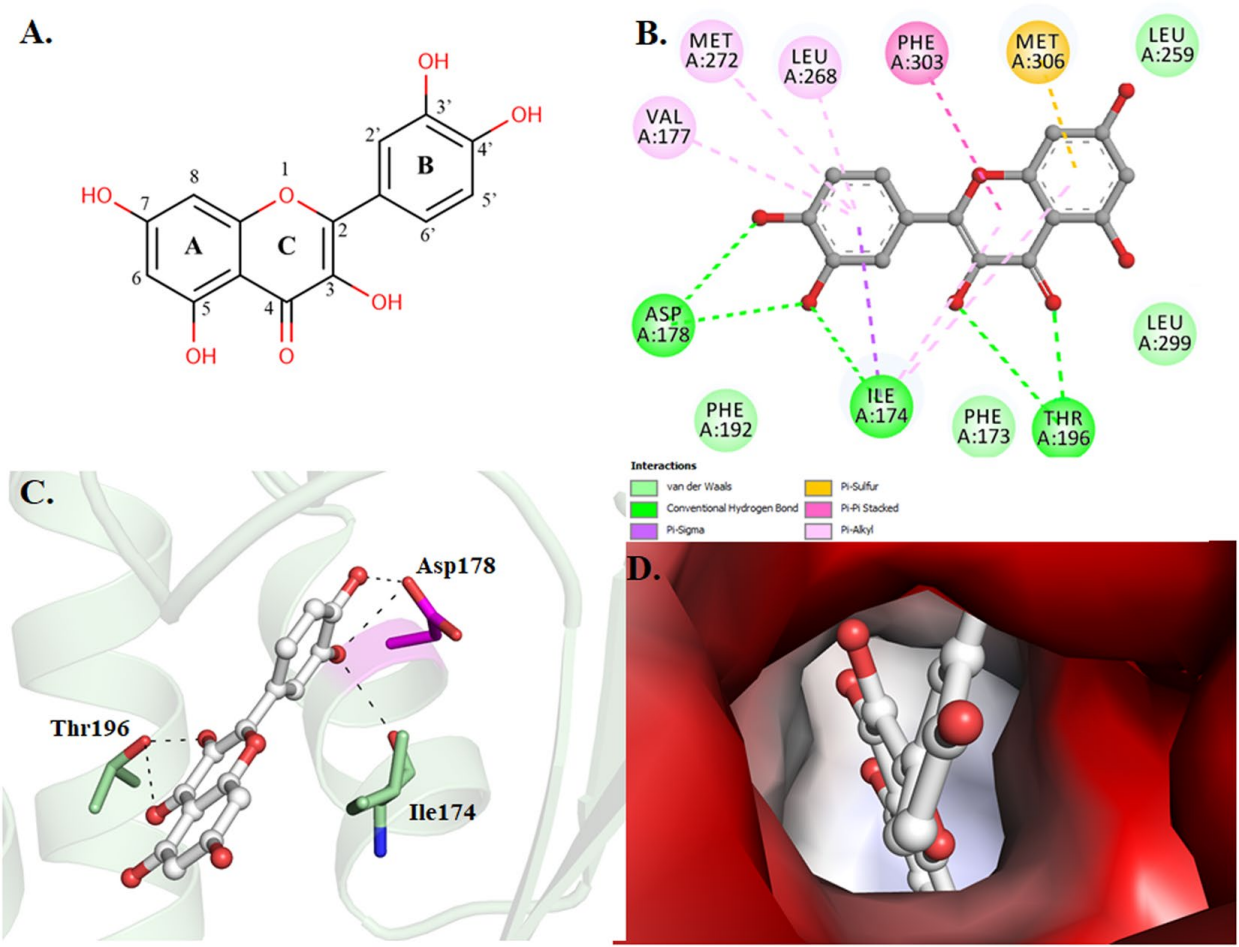
Interactions of quercetin with SphK1. (**A**) 2D structural representation of quercetin. The structure of quercetin is composed of three rings (A, B, and C) and five hydroxyl groups. (**B**) 2D structural representation of SphK1 residues interacting with quercetin. (**C**) Cartoon representation showing the docked quercetin interacting with the binding site residues of SphK1. (**D**) Surface potential view of SphK1 binding pocket occupied by quercetin.

Quercetin was found to occupied the distal end of the enclosed lipid pocket of SphK1 where the alkyl chain of sphingosine is found to bind, with the two phenolic rings A and C placed at the end of the pocket and the catechol ring B (3′,4′-dihydroxy group) directing toward the opening in the cleft between the N-terminal and the C-terminal domain (Fig. 4). The catechol ring forms three hydrogen bonds, two with Asp178 and one with Ile174, resulting in closed conformation for the lipid gate. At the other end, hydroxyl (−OH) and oxo (=O) groups of ring C are forming two hydrogen bonds with Thr196. The rest of the quercetin molecule forms several other non-covalent bonds such as π-Alkyl, π-Sigma and Van der Waals interactions with a similar set of non-polar residues (Phe173, Phe192, Leu259, Leu299, Val177, Leu268, Met272, Phe303 and Met306) that act to recognize the hydrophobic tail of the sphingosine (Fig. 5B and Table S2). Surface representation of SphK1 is indicating that the internal cavity of SphK1 is occupied by quercetin which is essentially binding with the substrate-binding pocket residues with an appreciable affinity (Fig. 5D). Hence, it could be speculated that quercetin bound at the catalytic site via interaction between its catechol group and the active site residue of SphK1 (Asp178) though hydrogen bond, and other stabilizing non-covalent interactions hinders the sphingosine accessibility, eventually leading to inhibit the activity of SphK1. Overall, the interaction analysis suggests that quercetin acts as a competitive inhibitor of sphingosine instead of being an ATP competitor like most of the kinase inhibitors.

### Structural changes in SphK1 upon quercetin binding

MD simulations have been utilized to get insight into the structural dynamics and functional mechanism of a protein-ligand complex. It has been become a widely used approach to describe the dynamics of the binding prototype of a small compound with a protein in explicit solvent environment^55^. Here, we performed all-atom MD simulation of SphK1 in the free state, and quercetin-bound SphK1 complex to evaluate the conformational dynamics, stability and interaction mechanism of quercetin with SphK1. Analysis of MD trajectories provides a detailed mechanistic insight into how quercetin binding affects the structural and dynamic behaviour of SphK1. It reveals that the quercetin plays a key role in stabilizing the structural conformation of SphK1. We calculated the potential energy of SphK1 before and after quercetin binding to ascertain the equilibration and stability of the complexes prior to MD analysis. The calculated potential energy of SphK1 before and after quercetin binding was found to be −890,000 kJ/mol and −880,713 kJ/mol, respectively. Several important MD parameters calculated for both the systems are given in Table 3.

**Table 3:** MD parameters calculated for SphK1 and SphK1-quercetin systems after 100 ns MD simulation.

Complex	RMSD (nm)	RMSF (nm)	*R*_g_ (nm)	SASA (nm^2^)	Kinetic energy (kJ/mol)	Enthalpy (kJ/mol)	Volume (nm^3^)	Density (g/l)
SphK1	0.37	0.13	1.96	145.61	145288	−744675	595.17	1028.44
SphK1-Quercetin	0.38	0.14	2.03	153.09	143877	−736800	589.42	1029.22

**Figure 6:**
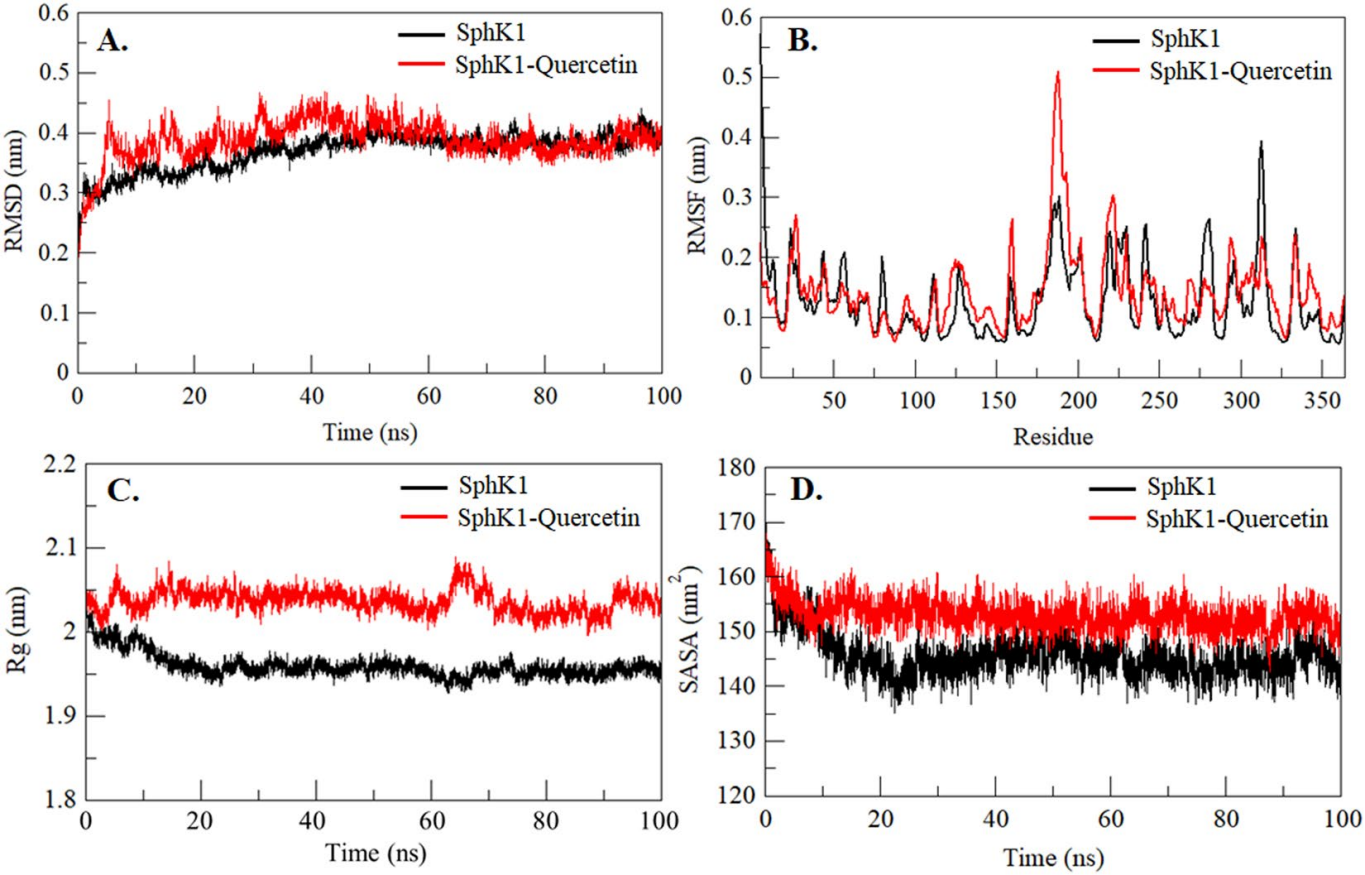
Structural dynamics of SphK1 before and after quercetin binding. (**A**) RMSD plot of SphK1 as a function of time. (**B**) Residual fluctuations (RMSF) plot of SphK1 and upon quercetin binding. (**C**) Time evolution of radius of gyration (*R*g). (**D**) SASA plot of SphK1 as a function of time. The values were obtained from the 100 ns MD simulation time scale. Black and red represents values obtained for free SphK1 and SphK1-quercetin complex, respectively.

A small molecule can cause large conformational changes in a protein structure after binding to its active pocket^56–59^. Calculating Root-mean-square deviation (RMSD) is one of the most widely used approaches to evaluate the structural deviation and conformational changes in a protein^60^. To validate the stabilization of SphK1 before and after quercetin binding, the RMSD of both systems were analyzed. Quercetin is found to stabilizes the global dynamics of SphK1 compared to free SphK1. During the analysis, an average of RMSD values for SphK1 before and after quercetin binding was found to be 0.37 nm and 0.38 nm, respectively (Table 3). Here we found no significant changes in RMSD values which suggests that the binding of quercetin stabilizes the structure and leads to less conformational changes from the native conformation of SphK1 (Fig. 6A). But, in RMSD analysis of the SphK1 flap aa 170–180, it was observed that the quercetin-bound SphK1 exhibits significant structural deviation (Fig. S4A). Here, it was found that, as compared to the free SphK1, quercetin-bound SphK1 fluctuates considerably and has the least RMSD throughout the simulation (Fig. S4A).

To see the residual movements and structural flexibility, the average fluctuation of each residue was evaluated and plotted as RMSFs in SphK1 before and after quercetin binding (Fig. 6B). We found several random fluctuations in the movement of each residue of SphK1 which were found to be minimized upon quercetin binding as depicted in the RMSF plot at region spanning from N- terminal to C- terminal. However, several increasing residual fluctuations were also seen upon quercetin binding, especially in the region spanning from amino acid residues 180 to 191 which overlaps with ATP-binding site residues (Arg185 and Arg191) (Figs. 4 and 6B). This suggests that structural flexibility induced by quercetin at ATP-binding pocket can also be presumed as a reason for reduced SphK1 activity along with the hindrance imparted by it for substrate binding as concluded from docking analysis. While in case of the flap of 170 to 180 residues that possess the substrate-binding site Asp178, RMSFs were found to be minimized reflecting significant conformational changes in SphK1 binding pocket upon interaction with quercetin (Fig. S4B). The fluctuation of substrate binding site Asp178 remains nearly constant and not changed significantly upon quercetin binding. However, increased fluctuation has been observed in Ile17, a functionally important residue of the substrate-binding lid of SphK1, upon binding to quercetin (Fig. S4B). The analysis shows that as compared to the free SphK1, quercetin-bound SphK1 fluctuates considerably throughout the simulation.

Radius of gyration (*R*_*g*_) is an important parameter of a protein accompanying with its overall conformational shape. It has been widely used to analyze the stability and conformational changes of a protein. The average *R*_*g*_ of free SphK1 and quercetin-bound SphK1 was calculated and found as 1.96 nm and 2.03 nm, respectively. Although the *R*_*g*_ plot shows little higher value in case of quercetin-bound SphK1, no significant structural switching was observed during the entire simulation (Fig. 6C). The protein attained a stable value of *R*_*g*_ equilibrated throughout the simulation. However, we observed significant changes in *R*_*g*_ of flap170–180 residues with decreased value, but no conformational shift was found suggesting least structural deviation in SphK1 upon quercetin binding (Fig. S4C).

Solvent Accessible Surface Area (SASA) is the area of a protein that is directly accessible to the surrounding solvents^61^. An average of SASA values for free SphK1 and quercetin-bound SphK1 were calculated and found to be 145.61 nm^2^, and 153.09 nm^2^, respectively. We observed that the quercetin induces a little conformational change in SphK1 as depicted in increased SASA as compared to free SphK1 (Fig. 6D). In contrast, we observed a little decrement in SASA of the flap of residues 170 to 180 when in complexed with quercetin (Fig. S4D). In overall observation of flap 170 to 180, we found increased dynamics of SphK1 due to the binding of quercetin (Fig. S4).

### Interaction analysis of SphK1-quercetin complex

**Figure 7:**
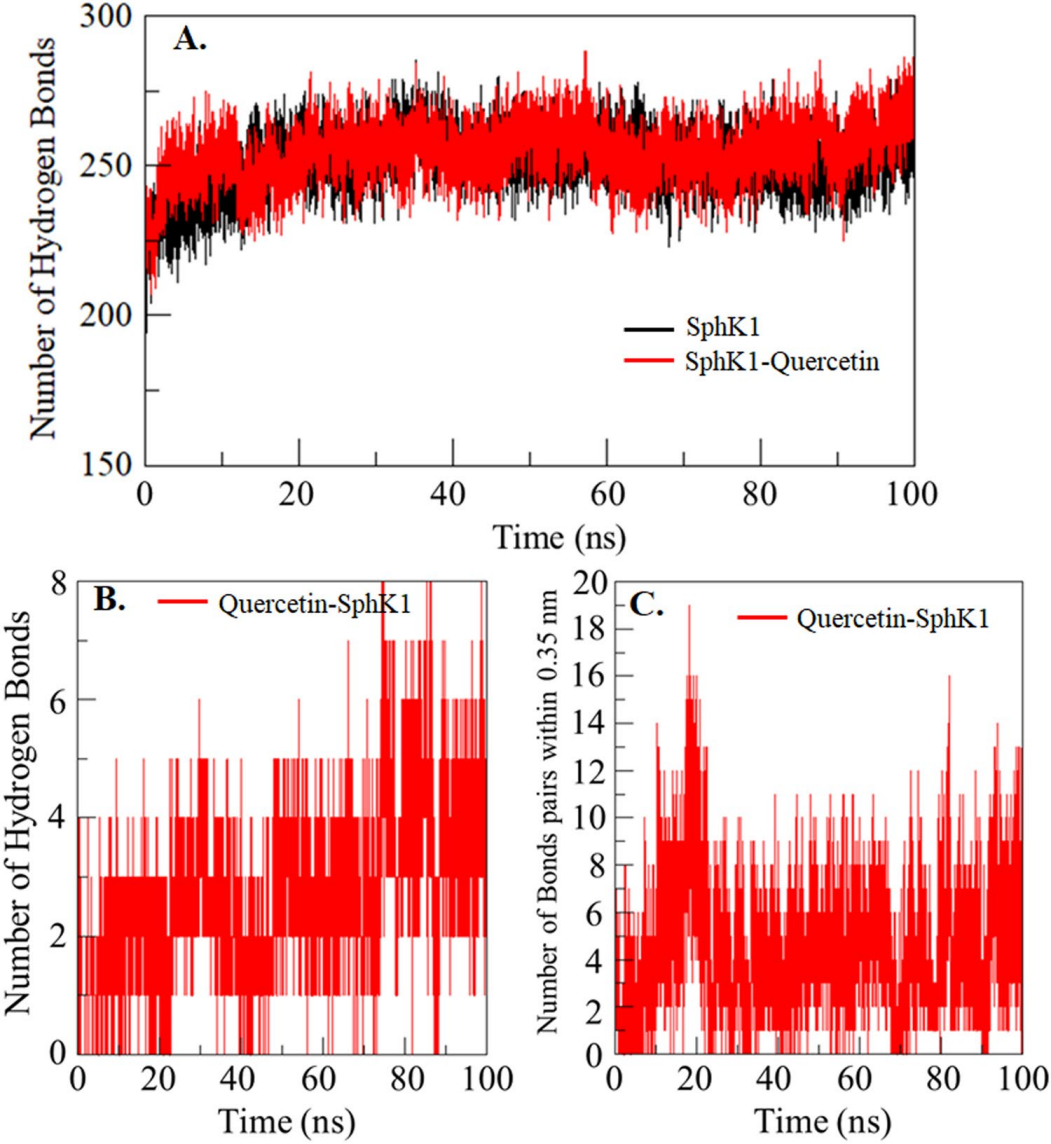
Time evolution and stability of hydrogen bonds formed. (**A**) Intramolecular in SphK1 and (**B**) Hydrogen bonds between quercetin and SphK1. (**C**) All bonds pairs within 0.35 nm between quercetin and SphK1.

Hydrogen bonding in a protein structure is a fundamental aspect of conformational stability^62,63^. The analysis of hydrogen bonds formed between a small molecule and a protein can be utilized to get insight into the stability of the complex which can provide directionality and specificity of protein-ligand interaction^62^. To assess the stability of SphK1 structure before and after quercetin binding, the intra-protein hydrogen bonds paired within 0.35 nm during the simulation were calculated as 251 and 254, respectively (Fig. 7A). An average of conventional hydrogen bonds between quercetin and SphK1 was also calculated with the stability of 3 bonds formed throughout the simulation. The quercetin was found to bind in SphK1 binding pocket with 4–5 conventional hydrogen bonds with higher fluctuation, and 3–4 hydrogen bonds with the least fluctuation (Fig. 7B). An average of bonds paired within 0.35 nm between quercetin and SphK1 was calculated 5 in number (Fig. 7C).

### Time evaluation of secondary structure changes in SphK1

**Figure 8:**
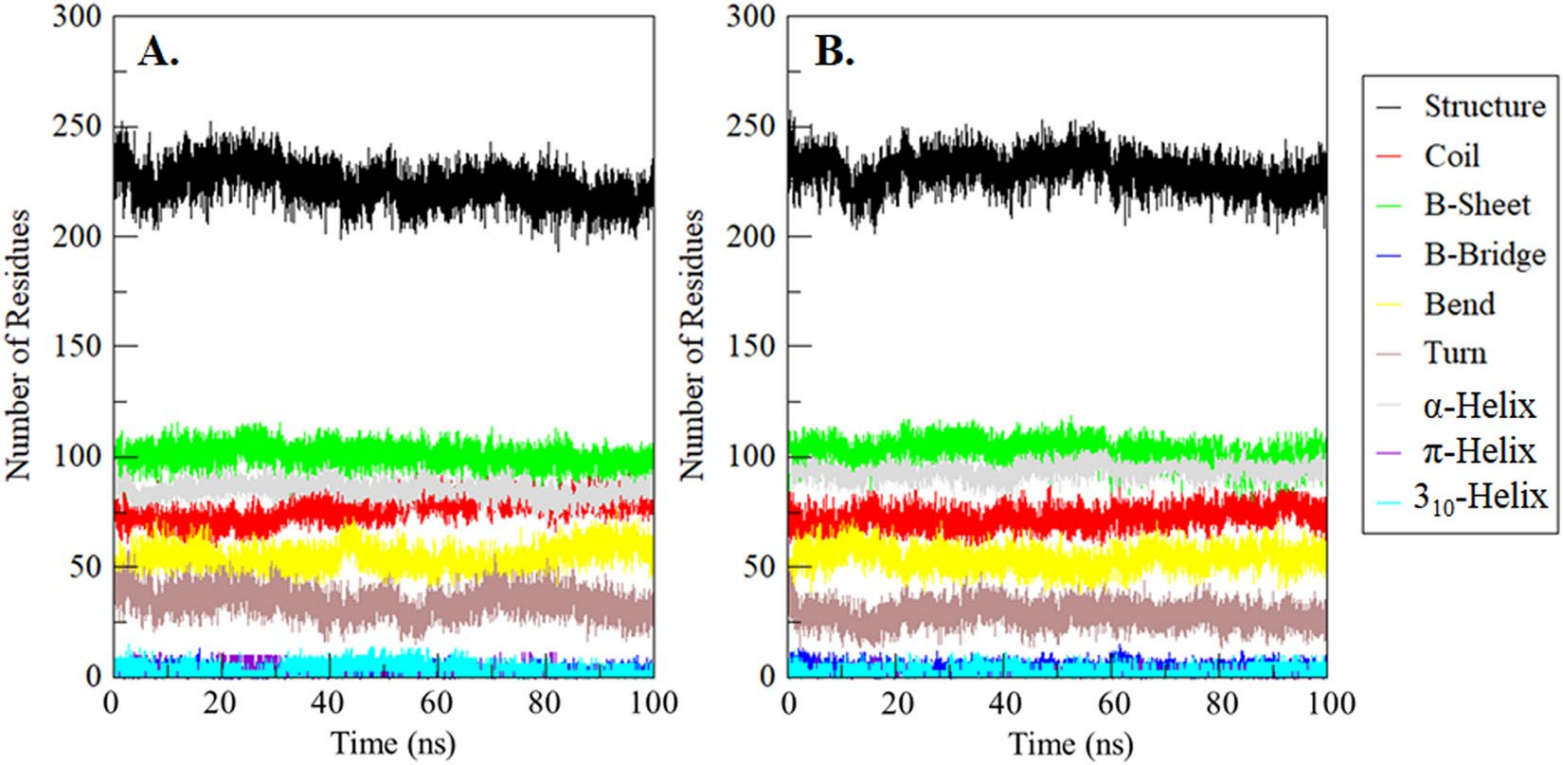
Time evaluation of the secondary structure content. (**A**) Free SphK1 and (**B**) SphK1 upon quercetin binding. *Structure = α-helix + β-sheet + β-bridge + Turn.

The time evaluation of changes in the secondary structure content of SphK1 before and after quercetin binding was calculated and plotted after the completion of the 100 ns simulation. The analysis showed that the structural elements i.e. α-helix, β-sheet and turn in SphK1 remain constant and equilibrated throughout the simulation with least fluctuation (Fig. 8A). Here, the average number of residues participating in secondary structure content of SphK1-quercetin complex was found to be a little increased as a result of the formation of α-helix and β-bridges and a little decrease in coil and turn as compared with free SphK1 (Fig. 8B and Table S3). We found no significant changes in the secondary structure content of SphK1 before and after quercetin binding.

### Principal component and free energy landscape analysis of SphK1

**Figure 9:**
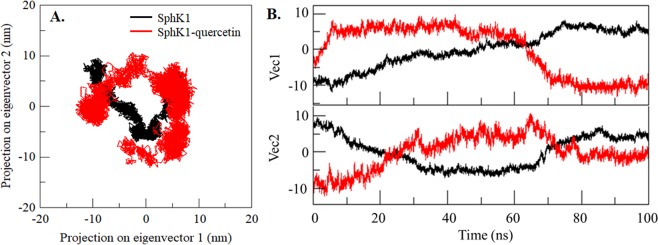
Principal Component Analysis. (**A**) 2D projections of trajectories on eigenvectors showed different projections of SphK1. (**B**) Projections of trajectories on eigenvectors with respect to time. Black and red represent values obtained for free SphK1 and SphK1-quercetin complex, respectively.

Principal component (PC) analysis is an extensively used method to get insight into the global motion of a protein as they formed their specific function by collective motion of their atoms. It identifies the essential modes representing the major part of the collective motions of the protein. We performed PC analysis to capture SphK1 motions before and after quercetin binding to evaluate the complex stability. We captured the significant motions of SphK1 via the top two eigenvectors, PC1 and PC2 before and after quercetin binding. We plotted the two-dimensional projection of the ensembles these PCs to compare the conformational space sampling by free SphK1 and quercetin-bound SphK1 (Fig. 9A). We can see the regions explored both the systems overlap with a notable difference in conformational sampling between the free and quercetin-bound states of SphK1 (Fig. 9A). This analysis is also showing consistency with RMSD and RMSF results, where the structural dynamics was found to be slightly increased in case of SphK1-quercetin complex. Here, we can see a wider cluster of the stable states of SphK1 in presence of quercetin, but, no overall switching was observed in the motion of quercetin-bound SphK1 (Fig. 9). The principal motions of SphK1 residues were visualized and analyzed by representing their PCs in porcupine plots. The principal motion of free SphK1 and SphK1-quercetin complex, along the direction of PC1 and PC2, are showing in Fig. 9C. We observed that in the free SphK1, loop regions of the SphK1 binding pocket experienced more flexibility as compared to the remaining part of the protein. Particularly, only the arm loop of the SphK1 binding pocket shows significant fluctuations whereas the remaining secondary structures exhibit minimal motion. The analysis of porcupine plots is suggesting that the binding of quercetin to SphK1 has allosterically influenced the structural dynamics of SphK1 (Fig. 9C).

**Figure 10:**
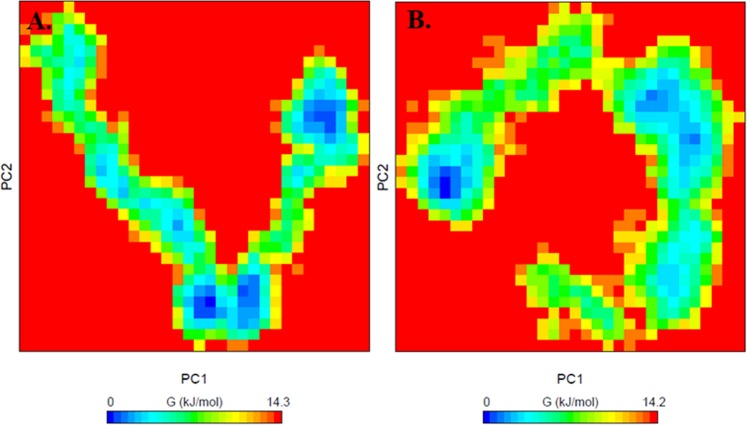
The Gibbs energy landscape obtained during 100 ns MD simulation for (**A**) free SphK1 and (**B**) SphK1-quercetin complex.

Further, to analyse the conformational behaviour of SphK1 before and after quercetin binding, the Gibbs free energy landscapes (FELs) were plotted using the first two PCs, PC1 and PC2. The FELs of SphK1 and SphK1-quercetin complex are shown in Fig. 10. SphK1, before and after quercetin binding shows 3–4 stable global minima confined within 2–3 basins as depicted from FEL. However, we can see a noticeable change in the conformational behaviour of SphK1 in-presence of quercetin which progress to different energy minima as compared to free SphK1 (Fig. 10).

## Discussion

The aberrant activation of signalling cascades is a frequent event in various types of human cancers^64,65^. Since protein and lipid kinases are key members of almost all signalling pathways, developing anticancer therapies targeting these central enzymes has always been a matter of choice among researchers^66–68^. To this end, SphK1 has emerged as a key enzyme as it regulates the sphingolipid rheostat responsible for determining cell fate. S1P generated by the catalytic action of SphK1 acts as pro-survival molecule and activates downstream targets involved in diverse pathological processes like cancer initiation, progression and inflammation. In fact, upregulation of SphK1 and its effector molecule S1P has been well established in various cancers and other human pathologies like pulmonary fibrosis, diabetes and Alzheimer’s disease. This makes SphK1 a potential drug target for the therapeutic intervention of diseases.

The identification and development of therapeutic molecules, targeting human kinase, capable of selectively killing the cancerous cells without being cytotoxic is of immense importance, and has received the growing interest of scientists globally^69–71^. Since ages, many plant-derived compounds or phytochemicals have been known to be effective against several human diseases including cancer owing to their anti-inflammatory, antioxidant, anticancer, antiviral and pro-apoptotic effects^36,37,72^. Additionally, they offer many advantages over synthetic drug molecules such as no or minimal side effects and being inexpensive. The immense importance of dietary polyphenols and other natural compounds encouraged us to investigate them as potential and effective inhibitors of SphK1.

Here, we studied the inhibitory potential of a series of plant-derived natural compounds. Among all, quercetin and capsaicin were found to possess best binding affinity towards SphK1 after the initial screening with molecular docking and fluorescence binding studies, and hence their role as SphK1 inhibitor was further evaluated. Quercetin is a polyphenolic flavonoid found in various fruits and vegetables whereas capsaicin, an active ingredient of chilli peppers, is a homovanillic acid derivative. The anti-carcinogenic, anti-angiogenic and anti-inflammatory effects of both of these dietary phytochemicals has been well established in the literature^73–78^. Recently, we have observed that quercetin and ellagic acid shows an excellent binding affinity to the pyruvate dehydrogenase kinase 3 and inhibits its enzyme activity^79,80^.

Fluorescence binding studies reveal that both quercetin and capsaicin binds SphK1 with high affinity, being maximum for quercetin; whereas other studied compounds do not show significant fluorescence quenching (Figs. 1 and S1). The binding parameters calculated form ITC measurements also suggested a strong interaction of both quercetin and capsaicin with SphK1 (Fig. 2 and Table 2). We further examined the ATPase activity of SphK1 in the presence of increasing concentration of compounds which shows an IC_50_ value of quercetin and capsaicin as 2.8 and 27.0 µM, respectively (Fig. 3 and Table 1). Enzyme inhibition assay further validate our fluorescence and ITC results.

Since quercetin was established as the most effective inhibitor of SphK1 among all screened natural compounds through a series of experiments, we tried to get mechanistic details of SphK1-quercetin interaction at molecular level by performing docking and MD simulation studies that could further facilitate our understanding of mechanism of inhibition by quercetin. Interestingly, a recent study by Zhang *et al*.^40^, illustrated that quercetin improves pulmonary fibrosis *in vivo* by inhibiting S1P/SphK1 signalling. This further encourages us to get mechanistic insights of the mode of inhibition of SphK1 by quercetin.

Molecular docking of quercetin with SphK1 was done to examine the binding pattern. Analysis of docking results revealed a strong binding affinity (−8.2 kcal/mol) of SphK1-quercetin complex (Table 1). The interaction analysis suggests that quercetin occupies the same position where the natural substrate D-sphingosine binds (Fig. 4). This suggests that quercetin decreases the substrate accessibility of SphK1 by acting as a competitive inhibitor that ultimately leads to enzyme inhibition. The SphK1-quercetin complex was stabilized by 3 hydrogen bonds as well as π-π interactions with the residues of substrate binding pocket (Fig. 5B and Table S2).

The stability and conformational changes occurring in SphK1 upon quercetin binding was further assessed by 100 ns MD simulation studies. The values of RMSD, RMSF, *Rg* and SASA suggest that quercetin binding stabilizes the SphK1 structure without any significant conformation shift (Table 3). However, several random fluctuations can be seen at initial time, but no conformational switching was observed during the entire simulations. This analysis is suggesting a strong stability of SphK1 upon quercetin binding. Notably, RMSF plot shows substantial conformational flexibility in the ATP binding pocket region upon quercetin binding which could probably disrupt the stabilizing interactions required for the ATPase activity of SphK1(Fig. 6B). This further explains the inhibitory potential of quercetin as assessed by our results of ATPase inhibition assay. We also investigated the anti-proliferative potential of quercetin on liver and lung cancer lines viz., HepG2 and A549. Quercetin was found to inhibit the growth of cancer cells in a dose dependent manner with an IC_50_ value of 49.10 µM and 55.90 µM for HepG2 and A549 cells, respectively (data not shown). At the same time, quercetin did not impart any toxicity towards normal counterparts (HEK293 cells).

## Conclusions

In conclusion, this study indicates that quercetin and capsaicin act as potent inhibitors of SphK1; being quercetin as the best inhibitor by interacting directly with the substrate binding pocket. Hence, targeting SphK1 by these natural compounds can be a smart therapeutic approach to manage the clinical manifestations of cancer and other SphK1 associated human pathologies. Overall, our results encourage the use of dietary phytochemicals in the development of therapeutics against SphK1 and other disease related kinases.

## Supplementary information

Suppll_Materials
