# Supplementary material for: Evaluation of binding and inhibition mechanism of dietary phytochemicals with sphingosine kinase 1: Towards targeted anticancer therapy
Source: Sci Rep. 2019 Dec 10;9:18727. doi: 10.1038/s41598-019-55199-3 (PMC6904568; doi:10.1038/s41598-019-55199-3)
Supplement: Supplementary file 1 — Suppll_Materials [file 41598_2019_55199_MOESM1_ESM.pdf]

Evaluation of binding and inhibition mechanism of dietary phytochemicals with sphingosine kinase 1: Towards targeted anticancer therapy

Preeti Gupta[1], Taj Mohammad[1], Rashmi Dahiya[1], Sonam Roy[1], Omar Mohammed Ali Noman[2], Mohamed F. Alajmi[2,*], Afzal Hussain[2] and Md. Imtaiyaz Hassan[1,*]

[1]Centre for Interdisciplinary Research in Basic Sciences, Jamia Millia Islamia, Jamia Nagar, New Delhi 10025, INDIA

[2]Department of Pharmacognosy College of Pharmacy, King Saud University, Riyadh, Saudi Arabia.

**To whom all correspondence should be addressed,*

Md. Imtaiyaz Hassan, Ph.D. FRSC., FRSB.
Centre for Interdisciplinary Research in Basic Sciences
Jamia Millia Islamia, Jamia Nagar
New Delhi 10025, INDIA
Cell: +91-9990323217
E-mail: mihassan@jmi.ac.in

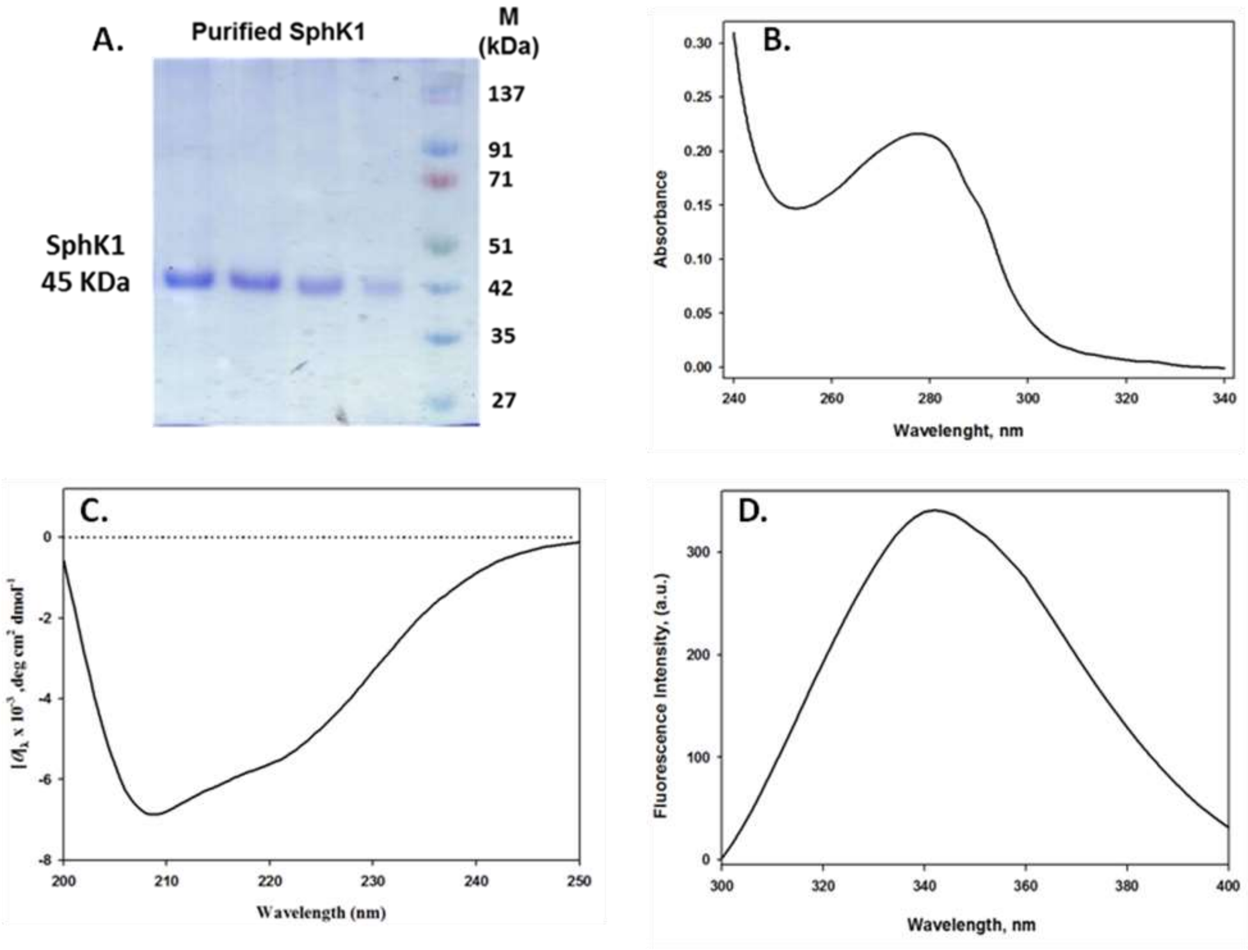

Figure S1: (A) SDS-PAGE profile showing single band of SphK1 at 45 kDa. **(B)** UV-Absorption spectra of SphK1 in the range of 240-340 nm. **(C)** Far-UV circular dichroism spectra of SphK1 showing proper folding (α/β fold) of purified protein. **(D)** Intrinsic fluorescence spectra of SphK1.

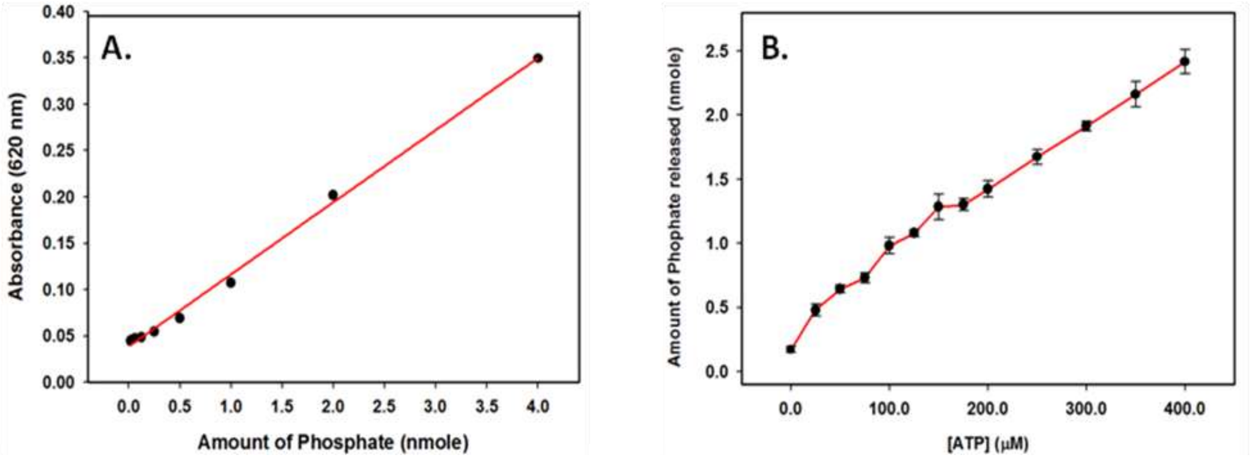

Figure S2: (A) Phosphate Standard curve. It was used to calculate amount of inorganic phosphate released by the catalytic action of SphK1. **(B)** ATPase assay of SphK1 showing the kinase activity with increasing amount of ATP.

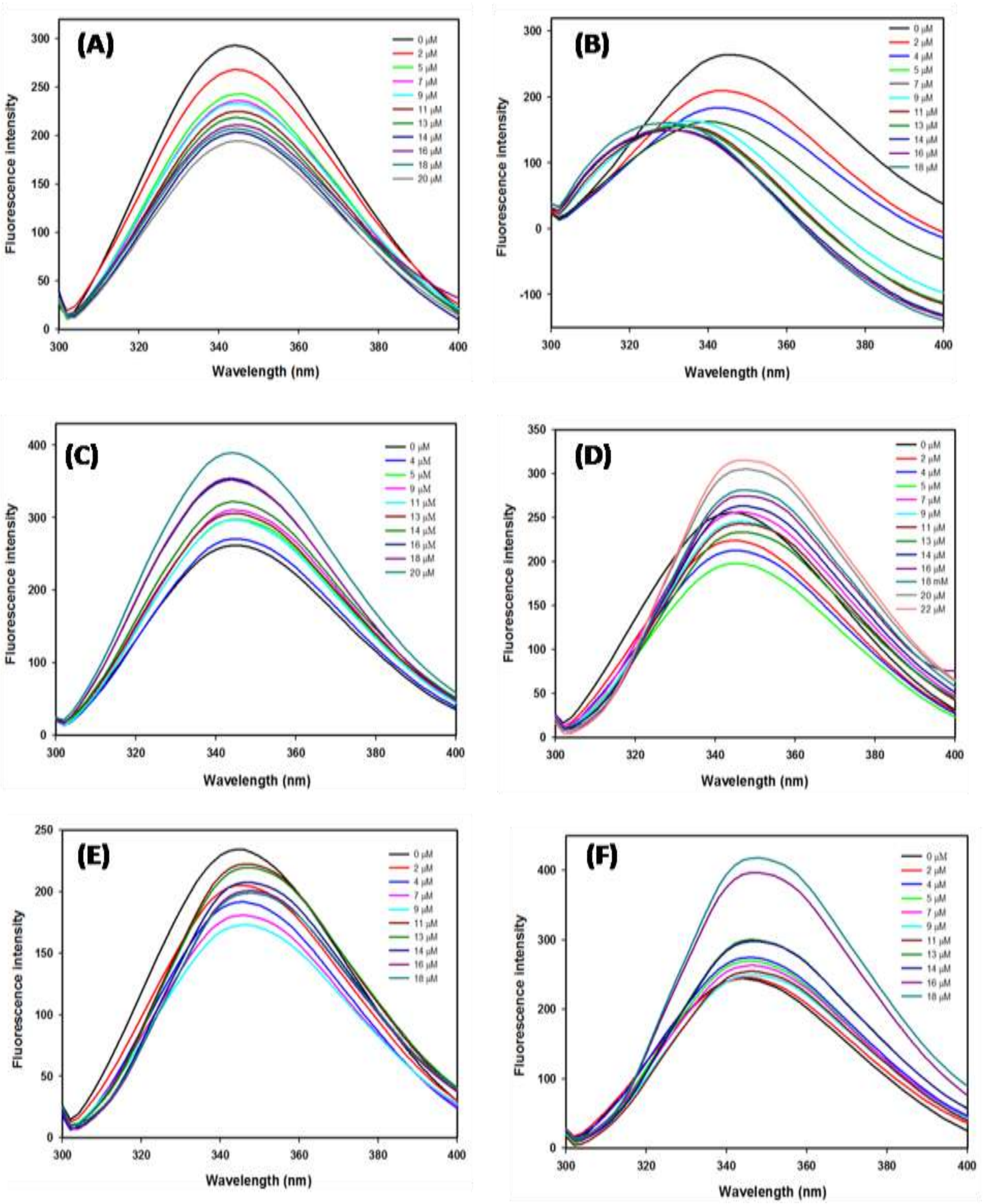

Figure S3: Fluorescence emission spectra of SphK1 (4 µM) with increasing concentrations of different natural compounds. **(A)** Ursolic acid **(B)** DL-α tocopherol acetate **(C)** Vanillin **(D)** Citral **(E)** Limonin **(F)** Simvastatin.

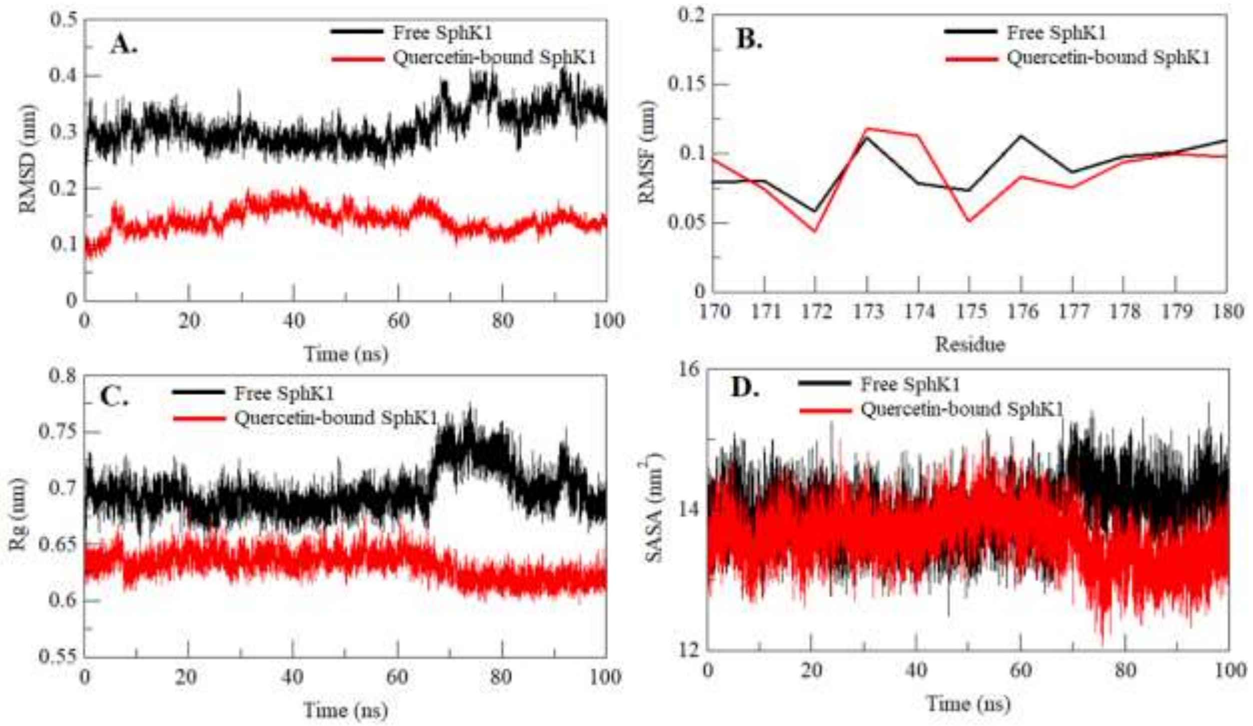

Figure S4: Structural dynamics of the flap 170-180 of SphK1 before and after quercetin binding. (**A**) RMSD plot of SphK1 as a function of time. (**B**) Residual fluctuations plot of SphK1 upon quercetin binding. (**C**) Time evolution of radius of gyration. (**D**) SASA plot of SphK1 as a function of time. The values were obtained from 100 ns MD simulation time scale.

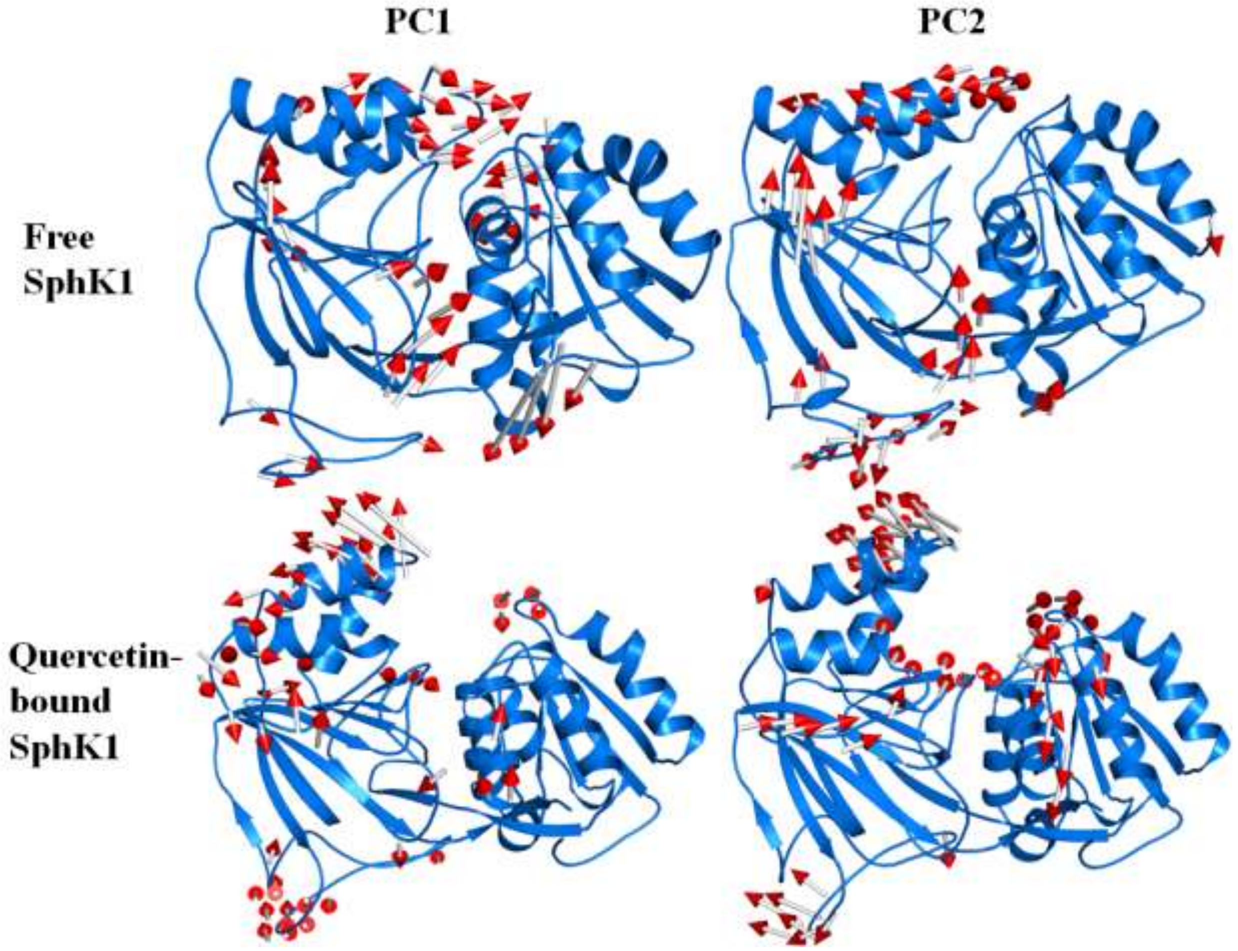

Figure S5: The porcupine plots representing the principal motions along the direction of PC1 and PC2, respectively are shown for free SphK1 and quercetin-bound SphK1. Red and white porcupines arrows represent motion according to the eigenvectors; length of the porcupine arrows represents the amplitude of motion.

Table S1. Binding parameters of all the natural compounds with SphK1 obtained from molecular docking and fluorescence binding studies.

Compound	Source	Chemical structure	***ΔG***[€] **(kcal/mole)**	[*]**Binding constant (*K*a), M-1**	[*]**Number of binding sites (*n*)**
Ursolic acid	Peels of fruits, as well as in herbs and spices	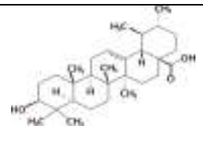	-7.8	3.13×10^3	1
Capsaicin	Chilli peppers	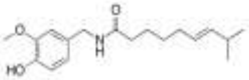	-8.1	1.53×10^4	1
DL-α tocopherol acetate	Soybean or rapeseed oil	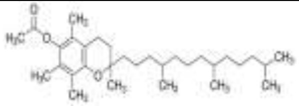	-6.6	NA	NA
Quercetin	Citrus fruits and green leafy vegetables	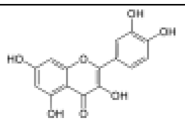	-8.2	4.38×10^5	1
Vanillin	Vanilla bean	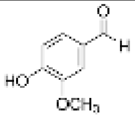	-5.3	NA	NA
Citral	Lemongrass oil	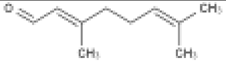	-6.1	NA	NA
Limonin	Citrus and other plants	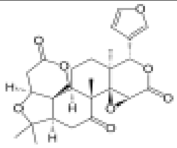	-7.6	NA	NA
Simvastatin	*Aspergillus terreus*	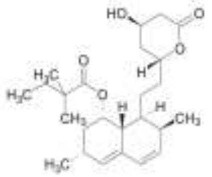	-7.0	NA	NA

[€]Binding affinity of the selected compounds with SphK1 predicted through AutoDock Vina. [*]Binding constant calculated from fluorescence studies. Some of the compounds do not show any fluorescence quenching in SphK1. Hence the values of K_a and n cannot be calculated in such cases and are depicted as not applicable (NA).

Table S2: Different interactions between quercetin and SphK1 interacting residues

S. No.	Interaction type	No. of Interactions	Participating Residues
1.	Hydrogen bonds	3	Ile174, Asp178 and Thr196
2.	van der Waals	4	Phe173, Phe192, Leu259 and Leu299
3.	Pi-Sigma	1	Ile174
4.	Pi-Alkyl	3	Val177, Leu268 and Met272
5.	Pi-Sulfur	1	Met306
6.	Pi-Cation	1	Phe303

Table S3: Percentage of residues participated in average structure formation.

Complex	Percentage of protein secondary structure							
	Structure*	Coil	β-sheet	β -bridge	Bend	Turn	α-helix	Other#
**SphK1**	62	21	28	1	15	9	24	2
**SphK1-quercetin**	64	20	29	1	15	8	26	1

*Structure = α-helix + β-sheet + β-bridge + Turn; #Other = π-Helix + 3_{10}-Helix
